# Predictors of Corporal Punishment during the COVID-19 Pandemic

**DOI:** 10.3390/pediatric16020026

**Published:** 2024-04-19

**Authors:** Robert D. Sege, Eliza Loren Purdue, Dina Burstein, Phyllis Holditch Niolon, Lori Lyn Price, Ye Chen, Elizabeth A. Swedo, Tammy Piazza Hurley, Kavita Prasad, Bart Klika

**Affiliations:** 1 Center for Community-Engaged Medicine, Institute for Clinical Research and Health Policy Studies, Tufts Medical Center, Boston, MA 02111, USA; robert.sege@tuftsmedicine.org (R.D.S.);; 2 Division of Violence Prevention, Centers for Disease Control and Prevention, Atlanta, GA 30329, USA; 3 Clinical and Translational Science Institute, Tufts Medical Center, Boston, MA 02111, USA; 4 American Academy of Pediatrics, Itasca, IL 60143, USA; thurley@aap.org; 5 School of Medicine, Tufts University, Boston, MA 02111, USA; 6 Prevent Child Abuse America, Chicago, IL 60604, USA

**Keywords:** corporal punishment, spanking, intimate partner violence, COVID-19

## Abstract

Although current policies discourage the use of corporal punishment (CP), its use is still widespread in the US. The objective of this study was to assess the proportion of parents who used CP during the pandemic and identify related risk and protective factors. We analyzed results of a nationwide cross-sectional internet panel survey of 9000 US caregivers who responded in three waves from November 2020 to July 2021. One in six respondents reported having spanked their child in the past week. Spanking was associated with intimate partner violence and the use of multiple discipline strategies and not significantly associated with region or racial self-identification. Parents who spanked sought out more kinds of support, suggesting an opportunity to reduce spanking through more effective parenting resources. Additionally, these results suggest that parents who report using CP may be at risk for concurrent domestic violence.

## 1. Introduction

Corporal punishment (CP) is broadly defined as “the use of physical force with the intention of causing pain but not injury, for the purpose of correction or control of the child’s behavior” [1] (p. 3). The use of CP in the United States has steadily decreased from an estimated 94% in the 1990s [2] to below 50% in recent studies [3,4].

Current research suggests that CP results in increased childhood aggression, criminal and antisocial behavior, externalizing behaviors, and risk of family violence in adulthood [5,6]. The results of a recent meta-analysis suggested that outcomes for children who experienced physical abuse and CP were similar [7].

Parental depression, substance abuse, other family stressors, and previous traumatic experiences have been associated with increased parental use of CP [8]. The American Academy of Pediatrics (AAP) released a policy statement in 2018 opposing the use of CP and provided guidance on more effective discipline strategies, including limit setting, redirecting, and setting clear goals and expectations for the future [8]. 

COVID-19 placed a great deal of stress on children, families, and communities [9]. Survey work by Lee and colleagues from early on in the pandemic shows that caregivers began experiencing elevated levels of social isolation, conflict, economic concerns, and mental health challenges, all of which are risk factors for CP [10,11]. Weekly survey data from the Rapid Assessment of Pandemic Impact on Development Early Childhood Project document that caregivers were experiencing significant material hardship, resulting in a “chain reaction” of personal mental health challenges, which then had an effect on child mental health [12]. As caregivers continued to experience the effects of pandemic stressors, there was concern this would result in harsh forms of discipline and abuse [13]. Some reports suggest that child abuse rates during the period of this survey did not increase, possibly due to economic support early on in the pandemic [14,15]. This study fills critical gaps in knowledge by examining self-report data collected from 9000 US caregivers throughout the early phase of the COVID-19 pandemic to understand the prevalence, and correlates (both risk and protective factors), of self-reported use of CP.

## 2. Materials and Methods

We analyzed data from a nationwide, cross-sectional survey conducted through an opt-in internet panel across three waves of 3000 caregivers/parents in November 2020, February 2021, and July 2021. The Measuring the Impact of Violence Against Children and Women During the Pandemic Questionnaire was developed via the collaborative effort of the AAP, the Centers for Disease Control and Prevention (CDC), Prevent Child Abuse America, and Tufts Medical Center [16]. As shown in Table 1, survey items were adapted from previously published instruments [10,17,18,19,20,21]. Additionally, the project team sought input on survey design from a nationwide partner council including pediatricians, parents, home visitors, and researchers. 

The survey was administered by the research data and analytics group YouGov [22]. Through various online recruitment methods, YouGov maintains a panel of respondents who have chosen to participate in online research activities. YouGov panelists go through a multi-step validation process and are verified through security questions. YouGov panelists are incentivized to complete surveys with the awarding of points that can be exchanged for rewards (e.g., Amazon gift cards). To identify a study sample, YouGov contacts a randomly selected cross-section of panelists to complete the survey. Using the demographic information provided by panelists on race and ethnicity, gender, age, income, education, and region of the country, YouGov weights the responding sample to a nationally representative sampling frame or profile derived from census data. To ensure that a diverse and representative sample is selected, YouGov directs panelists to surveys needing responses from someone fitting the panelist’s demographic profile and continues this process until a nationally representative sample population has been obtained. As demographic quotas are filled, those not filling the demographics still needed are screened out and redirected to other YouGov studies. 

For the present study, invitations were distributed via email to panelists who were between the ages of 18 and 95 years old, had children under 18 years of age living in their homes, and who speak and read English. All responses remained anonymous. No personally identifying information was collected. The Tufts Medical Center and AAP Institutional Review Boards determined that this was not human subject-based research. 

Survey items covered two conceptual domains: risk and protective factors and behavioral outcomes (Table 1). To assess risk and protective factors, caregivers were asked about a variety of experiences during the pandemic, including their current experiences with intimate partner violence (IPV), their history of adverse childhood experiences (ACEs), the types of stress-relieving activities that they utilized, social supports who they reached out to, recent feelings of closeness with or anger at children, and recreational activities that they had recently engaged in with their children. Caregivers were asked about their use of CP as a discipline method over the past week. 

Experiences with IPV were explored using questions derived from the CDC’s Violence Against Children and Youth survey [17]. Caregivers reported on their own experiences of physical and emotional IPV since the start of the COVID-19 pandemic (March 2020). Caregivers were also asked to report their own ACEs by selecting experiences that had occurred prior to their 18th birthday. 

Caregivers reported on activities that they used to deal with stress in the past month. Positive activities included yoga, meditation, prayer, exercise, watching television or other screen time, and reading. Negative activities included drinking alcohol, using tobacco products, using cannabis/marijuana, or using other substances/drugs (e.g., opioids, cocaine, LSD, etc.). Note that these responses including substance use only, and positive responses did not necessarily indicate substance use disorder.

Accessing social supports was assessed using a question derived from the Responses to Stress Questionnaire (RSQ) from Vanderbilt University’s Stress and Coping Research Laboratory [18]. Caregivers reported who they had reached out to for assistance in the past month. Feelings of anger at their child(ren) were measured using a question derived from the National Survey of Children’s Health [19], which asked about the frequency of caregivers feeling angry with their child(ren) in the past week. 

Caregivers were asked about feelings of closeness to and recreational activities with their child(ren) through questions derived from the Parenting in Context Research Lab survey [10]. Questions about recreational activities asked what kinds of activities caregivers had engaged in with their child(ren) in the past seven days. Caregivers also reported how close they felt to their child(ren) since the start of the COVID-19 pandemic (March 2020). 

Caregivers were asked how often they used different types of discipline strategies with their children in the past seven days using questions from the parent–child conflict tactics scale [20] and included in the Parenting in Context Research Lab survey [10]. Non-aggressive strategies included explaining to child(ren) why something they did was wrong, putting children in time-out, giving child(ren) something else to do, and sending the child(ren) to their room. Aggressive strategies included shouting, yelling, or screaming or threatening to spank, slap, or hit. Caregivers reporting that they spanked, slapped, or hit their child(ren) were categorized as reporting “spanking”. Analyses were limited to those respondents who provided information about discipline in the household and answered the question about spanking. Details of all answer options can be seen in Table 1.

### Analysis

Analyses were performed on the combined dataset across all waves. There were a total of 8550 respondents (weighted); 449 respondents (5.3%) had missing data on discipline in the home and were not included in analyses (5.3%). Descriptive statistics are reported as means and standard deviations or frequencies and percentages. 

Logistic regression analysis modeling the association between child and family demographics and characteristics and the use of spanking was performed using variables chosen based upon prior research, theory, and clinical observations. Variables were converted into categorical variables with dichotomous (yes or no) or multiple levels (count or frequency above a certain value) based on the question features. The selected variables were entered into the multivariable model simultaneously (current IPV, any parent ACEs, the number of negative stress relievers used, the number of different social support domains, number of positive stress relievers, the number of non-aggressive discipline methods used, the number of aggressive discipline methods used, the number of recreational activities with children, anger with children, closeness with children, race, gender, region, the number of children, children’s ages), along with an indicator variable for wave. Model diagnostics checking for multicollinearity and outliers that would greatly affect regression results were assessed. Each record was associated with a sample weight in all analyses. Missing data for all variables were removed during regression analysis (Table 2). Analyses were performed in SAS 9.4; a *p*-value of less than 0.05 was considered statistically significant. Adjusted odds ratios reported in the results were drawn from the multivariable model, unless specified as univariate.

## 3. Results

A total of 8212 email invitations were sent out in wave 1 of the survey, followed by 9395 in wave 2 and 6035 in wave 3. After accounting for potential participants who were deemed ineligible, response rates were 46.76%, 87.73%, and 86.05%, respectively. One in six (n = 1412, 16.5%) respondents reported spanking their child in the past seven days, and 22.7% (1894) of respondents reported current experience of IPV. Among those respondents who spanked their child(ren), 64.5% also reported currently experiencing IPV compared to 14.7% who did not report spanking. Of caregivers reporting spanking, 78.1% also reported experiencing one or more childhood ACE compared to 55.0% who did not report spanking (Table 2). While current IPV was significantly associated with higher odds of spanking compared to respondents reporting no current IPV (adjusted odds ratio (aOR) 4.08, 95% confidence interval (CI) 3.38–4.92, *p* < 0.0001; Table 3), there was no significant evidence of parental ACEs with this same relationship (aOR, 1.01, 95% CI 0.83–1.23, *p* = 0.93; Table 3).

The odds of spanking were lower with increased use of negative stress relievers, both in those who reported using just one negative stress relief activity (aOR 0.74, 95% CI 0.61–0.90, *p* = 0.0032; Table 3) and those using multiple (≥2) negative stress relief activities (aOR, 0.73, 95% CI 0.58–0.92, *p* = 0.0081; Table 3). There was no significant association between number of positive stress relief activities and spanking (*p* = 0.26; Table 3).

Of caregivers who reported spanking, 49.2% also reported participating in three or more recreational activities with their child(ren) in the last seven days compared to 73.7% who did not report spanking (Table 2). Respondents reporting engaging in three or four or more family recreational activities showed significantly lower odds of spanking than respondents reporting no engagement in recreational activities (aOR, 0.34, 95% CI 0.21–0.54, *p* < 0.0001 and aOR, 0.20, 95% CI 0.12–0.31 *p* < 0.0001, respectively; Table 3).

Caregivers were asked to rank feelings about their level of closeness to their children since the start of the COVID-19 pandemic. Those who reported feeling quite close or very close to their children showed lower odds of spanking in the multivariable model compared to those who did not report feeling close or very close to their children (aOR, 0.78, 95% CI 0.65–0.94, *p* = 0.01; Table 3). Feeling angry with children one or more times per day in the past week was significantly associated with higher odds of spanking compared to those who did not feel angry daily in the past week (aOR, 2.02, 95% CI 1.70–2.4, *p* < 0.0001; Table 3). 

Respondents were asked whom they had reached out to for help or assistance in the past month. Options included members of the family, friends, and community each representing a domain of support. Of caregivers who reported spanking, 75.0% had reached out for help from at least one domain compared to 44.1% of those who did not spank. These caregivers who reported spanking were also more likely to reach out to more domains for help, with 21.3% accessing three or more domains compared to 11.7% of caregivers who did not spank (Table 2). Those who reported spanking had higher odds of reaching out to more domains for help. Those reporting reaching out to one or more supports showed significantly higher rates of reporting spanking compared to caregivers who did not access any supports, with highest odds of reporting spanking being among those who reached out to three or more domains for help (aOR, 2.20, 95% CI 1.72–2.81, *p* < 0.0001; Table 3). 

Caregivers who reported spanking also used other discipline strategies. Over half (61.4%) of those who reported spanking also used all four non-aggressive discipline strategies, in comparison to 12.3% of caregivers who did not report spanking. Most (80.1%) caregivers reporting spanking also used the other two aggressive discipline strategies compared to only 9.7% who did not report spanking (Table 2). Caregivers reporting using 2–4 non-aggressive discipline methods in the past week had significantly higher odds of spanking compared to those using 0-1 non-aggressive discipline methods (aOR, 7.07, 95% CI 5.09–9.82, *p* < 0.0001; Table 3). Respondents who reported using aggressive discipline methods also had significantly higher odds of reporting spanking compared to those who reported not using any negative discipline methods (aOR, 14.5, 95% CI 10.77–19.51, *p* < 0.0001; Table 3). 

Respondents with one or more young child (aged 1–4) in the home had higher odds of reporting spanking compared to those without a young child in the home in the univariate model (OR, 1.89, 95% CI 1.68–2.13, *p* > 0.0001; Table 3). Child age was not included in multivariable model. Spanking was also associated with the number of children in the home: those with three children at home had decreased odds of reporting spanking compared to those with only one child at home (aOR, 0.61, 95% CI 0.43–0.88, *p* = 0.0075; Table 3), while families with four or more children did not show a significant association. Male respondents had higher odds of reporting spanking than female respondents (aOR, 1.73, 95% CI 1.47–2.05, *p* < 0.0001; Table 3). Race was not significantly associated with the reporting of spanking (Table 3).

## 4. Discussion

The first year of the pandemic led to dramatic changes in family life. Many of these changes were stressful for families [10]. This study examined parental and environmental factors related to the self-reporting of spanking. Our results indicate that one in six (16.5%) respondents reported spanking their child in the past seven days. 

IPV was strongly associated with spanking. If caregivers reported experiencing IPV in their current relationship, they were four times more likely to report spanking their children than those not reporting IPV, consistent with prior research [23]. Based on our results, pediatricians and other providers might ask about intra-familial violence when caregivers report the use of CP. 

Higher odds of spanking were also associated with other factors: male caregivers were more likely to report spanking than female caregivers. The presence of a child under age five was associated with spanking, consistent with findings that young children are more likely to be spanked than older children [24]. The race/ethnicity of the caregiver was not associated with reported spanking, suggesting that racial differences noted in a widely cited 1995 survey may no longer be significant [3,4]. 

After adjustment for other factors, parental ACEs were not significantly associated with spanking. Other variables included in the model associated with parental ACEs attenuated the relationship between parental ACEs and spanking seen in the univariate analysis. Further research is needed to investigate this relationship. 

Surprisingly, caregivers who reported engaging in negative stress-relieving behaviors had roughly 25% lower odds of reporting spanking than caregivers who reported not engaging in any negative stress-relieving behaviors. The prior literature demonstrates that while substance use disorder is consistently related to physical child abuse, the association between any substance use and physical aggression is less well established [25,26]. Engaging in positive stress-relieving behaviors did not result in either significantly lower or higher odds of reported spanking. The vast majority of caregivers did engage in some positive individual stress-relieving activities (fewer than one in five said they engaged in none of these), which may explain the lack of significant association between positive activities and spanking. 

Several aspects of caregiver–child dynamics were associated with decreases in reports of CP. Caregivers who reported three or more recreational activities as a family had significantly lower odds of reporting spanking than those with none. Those who reported feeling close to their children and those who had not felt angry at their children in the last week were significantly less likely to report spanking. Correlational data cannot discern whether these attributes reduced the use of CP, whether CP itself disrupted these markers of safe stable nurturing relationships, or whether unmeasured child or parent attributes contributed to both these attributes and the use of corporal punishment. For example, the child’s temperament or temperamental mismatch could be an important factor. Further research might explore the relationship between family recreational activities, relational health, and the prevention of CP. 

Caregivers who reported reaching out to more domains of support were more likely to report spanking in the last seven days than those who did not do so. Caregivers who reported reaching out to three or more support domains in the last month had double the odds of reporting spanking in the last week than those who did not seek support. Possibly, these respondents sensed their own parenting-related stress and both reached out for help and spanked their children. This finding represents an area of potential intervention, as caregivers who use corporal punishment may be open to utilizing resources for support. Future research could also investigate the temporality of reaching out for help and how that relates to CP.

Caregivers who reported spanking in the last week also reported trying multiple ways to manage their children’s behavior, both aggressive and non-aggressive, consistent with the view that many caregivers use spanking as a last resort [8]. 

Taken together, these results have potential implications for pediatricians and other care providers. (1) When parents disclose spanking, these results suggest a high risk for intimate partner violence in the home, (2) Parents and other caregivers who use CP have also tried other methods of discipline. Providers might ask parents and other caregivers about their behavioral goals for their children, what they have already tried, and offer additional support when a recommended strategy has not been effective. Key components of effective parenting programs focus on the use of positive reinforcement and nonviolent discipline techniques [27]. Many communities may offer evidence-based parent education programs that can help with behavior management [28].

### Limitations

Cross-sectional surveys do not support causal inference. Further research is required to examine the relationship between spanking and contextual stressors that were not included in this report, including family financial status, employment loss, and disruptions to child and family services. Additionally, due to low frequency, some variables with multiple answer choices were collapsed for analyses. Due to the specific sampling strategy and 7-day lookback period, it is not possible to use these data to determine whether the rate of CP use changed during the pandemic. These results conform to secular trends towards the reduced use of CP [4]. Internet survey panels include self-selected volunteers who may not be fully representative of the target population. YouGov uses non-probability sampling, which allows the recruitment of respondents to match a target population represented in the U.S. Census; this method tends to produce biased estimates compared to probability-based sampling methods [29,30]. Post-stratification weights are applied by YouGov to compensate for this sampling approach, but concerns remain surrounding the reliability of internet panel data [31,32]. As with all survey data, self-reported behavior is subject to social desirability and recall biases. The surveys were conducted during the COVID-19 pandemic that may limit generalizability; however, ongoing geopolitical stressors may continue to affect family life in a similar manner. In addition to these general limitations, we did not perform a formal psychometric analysis of this survey. Although items were drawn from validated surveys where possible, we were not always able to include complete validated scales. Finally, it is possible that caregivers may have engaged in other positive or negative stress relivers or aggressive or non-aggressive discipline methods that were not captured by the survey here.

## 5. Conclusions

During the first year of the pandemic, less than one in six caregivers reported spanking their children in the past week. Those who engaged in multiple recreational activities with their children, felt close to them, and had only low levels of anger were less likely to report spanking in the last week. Those caregivers who did report spanking in the past week were more likely to report current intimate partner violence, attempting to access multiple sources of support, and use of multiple discipline methods. These findings suggest there are opportunities to identify ways to support families that may reduce the likelihood of spanking.

## Figures and Tables

**Table 1:** Variable Domains and Sources.

Domain	Variable	Question Text	Response Options
Outcome	Spanking in the past week ^a^	Thinking about the last 7 days… How often, if at all, have you done each of the following when disciplining your child(ren) under 18? *Spanked, slapped, or hit your child(ren)* ^f^	i. A few times per day or more ii. Once per day iii. A few times in the last 7 days iv. Only once in the last 7 days v. Not at all in the last 7 days vi. Prefer not to say
Protective Factor	# of different domains from which requested help ^b^	Which, if any, of the following people have you gone to for help or assistance within the last month? Please select all that apply.	a. Spouse; b. Partner; c. My child(ren); d. Friend(s) e. Parent(s) f. Sibling(s) g. Other family member(s) h. Therapist or counselor i. Religious leader within my community or other figure from a faith/prayer/belief system j. Other (please specify) k. I have not gone to anyone for help or assistance in the past month
	# of positive stress relievers used	Which, if any, of the following activities have you done in order to deal with stress within the last month? ^f^	a. Yoga b. Meditation c. Prayer d. Exercise f. Reading
	# of non-aggressive discipline methods used ^a^	Thinking about the last 7 days… How often, if at all, have you done each of the following when disciplining your child(ren) under 18? *Explained to your child(ren); Put child(ren) in time-out; Sent child(ren) to their room; Gave child(ren) something else to do* ^f^	i. A few times per day or more ii. Once per day iii. A few times in the last 7 days iv. Only once in the last 7 days v. Not at all in the last 7 days vi. Prefer not to say
	# of recreational activities with children ^a^	Which, if any, of the following activities have you done with your child(ren) under 18 within the last 7 days? Please select all that apply.	a. Read books together b. Cooked together and enjoyed meals together c. Educational activities d. Told stories e. Went for walks f. Played sports/outdoor activities g. Watched TV or other media h. Played video games together i. Other j. I have not done any recreational activities with my children in the last 7 days
	Feelings of closeness with children during the COVID-19 pandemic ^a^	Since the Coronavirus (COVID-19) outbreak, (i.e., since March 2020), how close have you felt to your child(ren) under 18?	a. Not close at all b. Not very close c. Fairly close d. Quite close e. Extremely close
Risk Factor	Current IPV ^c^	Which, if any, of the following has a boyfriend/girlfriend, romantic partner, or spouse done to you prior to March 2020? Please select all that apply.	a. Slapped, pushed, shoved, shook, or intentionally threw something at you to hurt you b. Punched, kicked, whipped, or beat you with an object c. Choked, smothered, tried to drown you, or burned you intentionally d. Used or threatened you with a knife, gun, or other weapon e. Insulted, humiliated, or made fun of you in front of others f. Kept you from having your own money g. Tried to keep you from seeing or talking to your family or friends h. Kept track of you by demanding to know where you were and what you were doing i. Made threats to physically harm you j. None of these k. Prefer not to say
	Parent ACEs ^c,d,e^	Which, if any, of the following did you experience prior to your 18th birthday? Please select all that apply.	a. I lived with someone who was depressed, mentally ill, or attempted suicide b. I lived with someone who had a problem with drinking or using drugs, including prescription drugs c. I lived with someone who served time or was sentenced to serve time in a prison, jail, or other correctional facility d. My parents or guardians separated or divorced e. My parents or adults in my home slapped, hit, kicked, punched or beat each other up f. I was hit, beat, kicked, or physically hurt by a parent or an adult in my home g. I was sworn at, insulted, or put down by a parent or an adult in my home h. I experienced unwanted sexual contact (such as fondling or oral/anal/vaginal intercourse/penetration) with someone at least 5 years older than me or an adult i. I did not have enough to eat, had to wear dirty clothes, or had no one to protect or take care of me j. I felt that no one in my family loved me or thought I was special k. None of these l. Prefer not to say
	# of negative stress relievers used	Which, if any, of the following activities have you done in order to deal with stress within the last month? ^f^	g. Drinking alcohol h. Using tobacco products i. Using cannabis/Marijuana use j. Using other substances/drugs (i.e., opioids, LSD, cocaine, etc.)
	# of aggressive discipline methods used ^a^	Thinking about the last 7 days… How often, if at all, have you done each of the following when disciplining your child(ren) under 18? Please select one option on each row. *Shouted, yelled, or screamed at child(ren); Threatened to spank, slap, or hit child(ren)* ^f^	i. A few times per day or more ii. Once per day iii. A few times in the last 7 days iv. Only once in the last 7 days v. Not at all in the last 7 days vi. Prefer not to say
	Feeling angry with children ^d^	How often, if ever, have you felt angry with your child(ren) within the past 7 days?	a. A few times per day or more b. Once per day c. A few times in the last 7 days d. Only once in the last 7 days e. Not at all in the last 7 days

^a^ Question text adapted from the Stress and Parenting During the Coronavirus Pandemic Survey [20]. ^b^ Question text adapted from the Responses to Stress Questionnaire (RSQ) [18]. ^c^ Question text adapted from the Violence Against Children Survey (VACS) [17]. ^d^ Question text adapted from the National Survey of Children’s Health (NSCH) [19]. ^e^ Question text adapted from the Pediatric Early Adversity and Related Life Effect Screen (PEARLS) [21]. ^f^ Data from this question were used for multiple variable calculations; see italicized text and response options for details on variable derivation.

**Table 2:** Percentage of caregivers reporting spanking in past week by exposure variable ^a^.

Variable	Category	All N = 8550	Not Reported Spanking in Past Week (%) N = 7138	Reported Spanking in Past Week (%) N = 1412
Current IPV	0: No	6435 (77.3)	5957 (85.3)	478 (35.5)
	1: Yes	1894 (22.7)	1026 (14.7)	868 (64.5)
	Missing	222	155	67
Any parent ACES	0: No	3406 (41.3)	3113 (45)	292 (21.9)
	1: Yes	4841 (58.7)	3798 (55)	1043 (78.1)
	Missing	304	227	77
# of negative stress relievers used	0	5399 (63.1)	4591 (64.3)	808 (57.2)
	1	2009 (23.5)	1647 (23.1)	362 (25.6)
	2	798 (9.3)	643 (9)	155 (10.9)
	3	269 (3.1)	211 (3)	57 (4.1)
	4	77 (0.9)	46 (0.6)	30 (2.2)
	Missing	0	0	0
# of different domains from which requested help	0	4342 (50.8)	3990 (55.9)	353 (25)
	1	1936 (22.6)	1432 (20.1)	503 (35.7)
	2	1129 (13.2)	874 (12.2)	255 (18.1)
	3	686 (8)	501 (7)	185 (13.1)
	4	289 (3.4)	231 (3.2)	58 (4.1)
	5	117 (1.4)	81 (1.1)	36 (2.5)
	6	32 (0.4)	19 (0.3)	13 (0.9)
	7	14 (0.2)	6 (0.1)	8 (0.6)
	8	3 (0)	2 (0)	1 (0.1)
	9	1 (0)	1 (0)	0 (0)
	Missing	2	1	1
# of positive stress relievers used	0	1645 (19.2)	1366 (19.1)	279 (19.8)
	1	2131 (24.9)	1712 (24)	419 (29.6)
	2	2093 (24.5)	1742 (24.4)	351 (24.9)
	3	1485 (17.4)	1297 (18.2)	187 (13.3)
	4	793 (9.3)	689 (9.7)	103 (7.3)
	5	305 (3.6)	250 (3.5)	55 (3.9)
	6	98 (1.1)	81 (1.1)	17 (1.2)
	Missing	0	0	0
# of non-aggressive discipline methods used	0	1787 (20.9)	1777 (24.9)	10 (0.7)
	1	1649 (19.3)	1608 (22.6)	41 (2.9)
	2	1944 (22.8)	1742 (24.4)	202 (14.4)
	3	1413 (16.6)	1126 (15.8)	287 (20.5)
	4	1740 (20.4)	878 (12.3)	862 (61.4)
	Missing	7	7	0
# of aggressive discipline methods used ^b^	0	4419 (51.8)	4360 (61.2)	60 (4.3)
	1	2294 (26.9)	2076 (29.1)	218 (15.6)
	2	1810 (21.2)	690 (9.7)	1120 (80.1)
	Missing	22	14	8
# of recreational activities with children	0	432 (5)	367 (5.1)	65 (4.6)
	1	1034 (12.1)	624 (8.7)	410 (29)
	2	1117 (13.1)	874 (12.2)	243 (17.2)
	3	1347 (15.7)	1135 (15.9)	211 (15)
	4	1211 (14.2)	1065 (14.9)	146 (10.3)
	5	1054 (12.3)	937 (13.1)	117 (8.3)
	6	914 (10.7)	815 (11.4)	100 (7.1)
	7	826 (9.7)	752 (10.5)	74 (5.2)
	8	575 (6.7)	530 (7.4)	45 (3.2)
	9	41 (0.5)	39 (0.5)	2 (0.1)
	Missing	0	0	0
Male	0: No	4639 (54.3)	4022 (56.3)	617 (43.7)
	1: Yes	3912 (45.7)	3116 (43.7)	796 (56.3)
	Missing	0	0	0
Race	1: White	4797 (56.1)	4093 (57.3)	704 (49.8)
	2: Black	997 (11.7)	855 (12)	142 (10.1)
	3: Hispanic	1892 (22.1)	1450 (20.3)	442 (31.3)
	4: Asian	278 (3.2)	223 (3.1)	55 (3.9)
	5: Native American	117 (1.4)	93 (1.3)	24 (1.7)
	9: Other/multiple	470 (5.5)	424 (5.9)	45 (3.2)
	Missing	0	0	0
Region	Midwest	1716 (20.1)	1512 (21.2)	204 (14.4)
	Northeast	1443 (16.9)	1201 (16.8)	242 (17.1)
	South	3213 (37.6)	2715 (38)	498 (35.3)
	West	2179 (25.5)	1710 (24)	469 (33.2)
	Missing	0	0	0
Closeness with children during the COVID-19 pandemic	0: Not close/not very close/fairly close	1979 (23.1)	1421 (19.9)	558 (39.5)
	1: Quite close/very close	6572 (76.9)	5717 (80.1)	855 (60.5)
	Missing	0	0	0
Angry with children	0: A few times last 7 days, once in last 7 days, not in last 7 days	7003 (81.9)	6228 (87.3)	775 (54.9)
	1: At least once/day, last 7 days	1547 (18.1)	910 (12.7)	637 (45.1)
	Missing	0	0	0

Abbreviations: IPV, intimate partner violence; ACEs, adverse childhood experiences. ^a^ In total, 1412 (16.5%) of respondents reported using spanking in the past 7 days. A further 449 respondents chose not to provide any information about discipline in the home. The tables and analyses below are limited to respondents who provided information about discipline and responded to the question about spanking. There are 8642 observations in the dataset (unweighted), and 8550 are weighted. ^b^ Spanking is not included in the count of negative discipline methods used.

**Table 3:** Univariate and multivariable models of association of spanking by exposure variable ^a^.

		Univariate				Multivariable		
Variable	Category	N	OR (95% CI)	*p*-Value	Global *p*-Value	OR (95% CI)	*p*-Value	Global *p*-Value
Current IPV	Any IPV (current)	8451	10.38 (9.12, 11.83)	<0.0001		4.08 (3.38, 4.92)	<0.0001	
Any parent ACEs	Any ACEs	8386	2.92 (2.54, 3.35)	<0.0001		1.01 (0.83, 1.23)	0.9281	
# of negative stress relievers used	0	8642	Reference		<0.0001			0.0027
	1		1.22 (1.07, 1.40)	0.0037		0.74 (0.61, 0.90)	0.0032	
	≥2		1.49 (1.27, 1.75)	<0.0001		0.73 (0.58, 0.92)	0.0081	
# of different domains from which requested help	0	8640	Reference		<0.0001			<0.0001
	1		3.92 (3.37, 4.55)	<0.0001		1.53 (1.23, 1.91)	0.0001	
	2		3.32 (2.78, 3.96)	<0.0001		1.54 (1.20, 1.98)	0.0008	
	≥3		4.01 (3.38, 4.76)	<0.0001		2.20 (1.72, 2.81)	<0.0001	
# of positive stress relievers used	0	8642	Reference		<0.0001			0.2642
	1		1.20 (1.01, 1.42)	0.0335		0.83 (0.64, 1.08)	0.1570	
	2		1.01 (0.85, 1.2)	0.9499		0.96 (0.74, 1.26)	0.7913	
	3		0.70 (0.58, 0.86)	0.0006		0.84 (0.62, 1.14)	0.2743	
	≥4		0.84 (0.68, 1.03)	0.0865		1.07 (0.78, 1.48)	0.6627	
# of non-aggressive discipline methods used	0–1	8634	Reference					
	2–4		23.78 (17.93, 31.54)	<0.0001		7.07 (5.09, 9.82)	<0.0001	
# of aggressive discipline methods used ^a^	0	8620	Reference					
	1–2		35.11 (26.96, 45.72)	<0.0001		14.5 (10.77, 19.51)	<0.0001	
# of recreational activities with children	0	8642	Reference		<0.0001			<0.0001
	1		3.65 (2.72, 4.89)	<0.0001		1.12 (0.69, 1.80)	0.6572	
	2		1.59 (1.18, 2.15)	0.0024		0.63 (0.39, 1.01)	0.0561	
	3		1.06 (0.78, 1.44)	0.6954		0.34 (0.21, 0.54)	<0.0001	
	≥4		0.66 (0.50, 0.88)	0.0040		0.20 (0.12, 0.31)	<0.0001	
Race	1: White	8642	Reference		<0.0001			0.3193
	2: Black		0.97 (0.79, 1.17)	0.7300		0.95 (0.73, 1.25)	0.7150	
	3: Hispanic		1.78 (1.56, 2.03)	<0.0001		1.19 (0.97, 1.46)	0.0971	
	4: Other		0.98 (0.80, 1.20)	0.8393		0.99 (0.74, 1.32)	0.9487	
Sex	Yes to “Male”	8642	1.67 (1.49, 1.88)	<0.0001		1.73 (1.47, 2.05)	<0.0001	
Region	South	8642	Reference		<0.0001			0.0242
	Midwest		0.74 (0.62, 0.88)	0.0007		0.80 (0.63, 1.02)	0.0692	
	Northeast		1.10 (0.93, 1.30)	0.2829		0.97 (0.76, 1.24)	0.8083	
	West		1.52(1.32, 1.75)	<0.0001		1.18 (0.96, 1.46)	0.1158	
Closeness with children during the COVID-19 pandemic	Quite close/very close	8642	0.39 (0.34, 0.44)	<0.0001		0.78 (0.65, 0.94)	0.0104	
Anger with children	At least once/day, last 7 days	8642	5.58 (4.91, 6.33)	<0.0001		2.02 (1.70, 2.4)	<0.0001	
Children’s Ages ^b^	Age 1–4	8642	1.89 (1.68, 2.13)	<0.0001				
	Age 5–11		1.48 (1.32, 1.66)	<0.0001				
	Age 12–18		0.39 (0.34, 0.44)	<0.0001				
# of children	1	8642			<0.0001			0.0108
	2		1.23 (1.09, 1.4)	0.0011		0.97 (0.77, 1.21)	0.7633	
	3		0.80 (0.65, 0.98)	0.0278		0.61 (0.43, 0.88)	0.0075	
	≥4		1.06 (0.82, 1.38)	0.6618		0.94 (0.57, 1.55)	0.8146	

Abbreviations: IPV, intimate partner violence; ACEs, adverse childhood experiences; CI, confidence interval; OR, odds ratio. ^a^ Spanking is not included in the count, only threatening and yelling. ^b^ Each variable is coded as 1 if the household has at least one child within that age range and 0 otherwise. Child age is not included in multivariable model.

## Data Availability

Data will be made available to qualified investigators upon inquiry.
